# The Effects of Temperature on the Hydrothermal Synthesis of Hydroxyapatite-Zeolite Using Blast Furnace Slag

**DOI:** 10.3390/ma12132131

**Published:** 2019-07-02

**Authors:** G.U. Ryu, G.M. Kim, Hammad R. Khalid, H.K. Lee

**Affiliations:** 1 Department of Civil and Environmental Engineering, Korea Advanced Institute of Science and Technology, Guseong-dong, Yuseong-gu, Daejeon 305-701, Korea; 2 Center for Carbon Mineralization, Climate Change Mitigation and Sustainability Division, Korea Institute of Geoscience and Mineral Resources, 124 Gwahak-ro, Yuseong-gu, Daejeon 34132, Korea; 3 Civil & Environmental Engineering Department, King Fahd University of Petroleum & Minerals, Dhahran 31261, Saudi Arabia

**Keywords:** hydroxyapatite, zeolite, adsorbent, hydrothermal method, blast furnace slag

## Abstract

Blast furnace slag, an industrial by-product, is emerging as a potential raw material to synthesize hydroxyapatite and zeolite. In this study, the effects of temperature on the hydrothermal synthesis of hydroxyapatite-zeolite from blast furnace slag were investigated. Specimens were synthesized at different temperatures (room temperature, 50, 90, 120, or 150 °C). The synthesized specimens were analyzed qualitatively and quantitatively via X-ray diffraction (XRD), Fourier transform infrared spectroscopy (FT-IR), BET/BJH, and scanning electron microscopy/energy dispersive using X-ray analysis (SEM/EDX). It was found that the hydroxyapatite phase was synthesized at all the reaction temperatures, while faujasite type zeolite appeared in the specimens synthesized at 90 and 120 °C. Moreover, faujasite was replaced by hydroxysodalite in the specimens synthesized at 150 °C. Additionally, the crystals of the hydroxyapatite tended to become larger and total crystallinity increased as the reaction temperature increased.

## 1. Introduction

Hydroxyapatite (Ca_10_(PO_4_)_6_(OH)_2_) and zeolites (alumino-silicate materials represented by M_m_[Al_m_Si_n_O_2(m+n)_] _·_H_2_O where M represents alkali cations electrostatically bonded to the extra skeleton) have been used for various applications, i.e., adsorption and catalysis [1,2]. Specifically, they have been studied as adsorbents due to their high specific surface area and the ionic forces induced by the presence of exchangeable ions in their structures [3,4,5]. 

The adsorption performance of zeolites for cations is much higher than that for anions since the exchangeable ions in zeolites are usually alkali cations [6,7]. On the other hand, hydroxyapatite phases have high adsorption performance for both the cations and anions [4,6,8,9]. Cations are exchanged with calcium ions present in hydroxyapatite [6,10], while anions are exchanged with hydroxyl ions [4]. 

Hydroxyapatite and zeolites are normally synthesized by hydrothermal methods [11,12,13]. The reaction temperature for hydrothermal treatment is an important variable for the characteristics of the resulting products [14,15]. If the reaction temperature is not high enough, hydroxyapatite and zeolites cannot be synthesized, and it can result in a low specific surface area [14,16]. The formation rate of different crystals and the aspect ratio of hydroxyapatite crystals also get affected by the reaction temperature [17]. Similarly, an increase in the reaction temperature was favorable to synthesize zeolite crystals through a hydrothermal process [16]. 

In this study, blast furnace slag, which is a by-product of the iron industry, was used to synthesize hydroxyapatite and zeolite as main phases. Both of these materials were targeted, considering the fact that zeolites are efficient adsorbents for cations, and they can support hydroxyapatite to adsorb cations and anions simultaneously. Because of its low price and mineral composition, it has been used in various fields, especially the construction and environmental fields [1,18,19]. However, about 35% of the slag is still being wasted in Europe alone [20]. Owing to the chemical composition of slag, it can be used to synthesize hydroxyapatite and zeolites [12,21,22]. Recently, Khalid et al. [12] proposed a robust one-step hydrothermal treatment method to synthesize geopolymer-supported zeolite adsorbents using blast furnace slag and fly ash. Guo et al. [23] synthesized NaA-zeolite from blast furnace slag and studied their performance for removal of ammonium. Kuwahara et al. [1] and Li et al. [24] studied the effects of reaction time on the synthesis of zeolite and sodalite using Ti-bearing electric arc furnace slag.

Despite the high potential of slag utilization for the synthesis of these adsorbents, only two studies have been reported on the synthesis of hydroxyapatite using blast furnace slag [1,11]. Furthermore, it is known that the reaction temperature during hydrothermal treatment can significantly affect the resulting crystalline phases and total crystallinity [14,15,25]. Therefore, it is crucial to investigate the effects of the reaction temperature on the synthesis of hydroxyapatite using blast furnace slag, which has not been reported in the literature to the best of our knowledge. Hence, this study specifically focused on the investigation of effects of reaction temperature on the crystallinity, content, and dimension of synthesized hydroxyapatite and zeolite via the hydrothermal synthesis method. The specimens were synthesized at different reaction temperatures (room temperature, 50 °C, 90 °C, 120 °C, or 150 °C). The characteristics of the specimens were investigated by X-ray diffraction (XRD), Fourier transform infrared spectroscopy (FT-IR), and scanning electron microscopy/energy dispersive using X-ray analysis (SEM/EDX), while microstructural characteristics were investigated via BET/BJH method.

## 2. Materials and Methods 

### 2.1. Materials

The chemical composition of the blast furnace slag used in the present study was investigated by XRF; results are listed in Table 1. The slag was mainly composed of CaO, Al_2_O_3_, and SiO_2_ components, which are needed to synthesize hydroxyapatite and zeolites. 

### 2.2. Test Methods

The synthesis process for hydroxyapatite-zeolite consisted of three steps. In the first step, 25 g of slag and 100 mL of 1.77 M phosphoric acid (H_3_PO_4_, Samchun Pure Chemical, Pyeongtaek, Korea) solution were stirred at 350 rpm using a magnetic stirrer at ambient temperature for 2 h. In this step, the molar ratio of Ca/P was fixed at 1.67 considering the theoretical Ca/P ratio of hydroxyapatite is 1.67. In the second step, 350 mL of 2 M sodium hydroxide (NaOH, Daejung Chemical & Materials Co., LTD, Goryeong, Korea) solution was added to the prepared suspension. Mixtures were stirred at 450 rpm for 4 h at their respective temperatures (such as room temperature, 50, 90, 120, or 150 °C) on the bottom of the beakers and specimens were denoted by HZ-R, HZ-50, HZ-90, HZ-120, and HZ-150, respectively. The mixture containers were fully covered with an aluminum foil to prevent the mixtures from water evaporation during stirring. In the last step, the prepared suspensions were transferred to sealed Teflon bottles and heated for 24 h in an oven again at their respective temperatures (i.e., room temperature, 50, 90, 120, or 150 °C). After the last step, white powders were obtained. The obtained powders were washed several times with distilled water, followed by filtration and drying. 

X-ray diffraction (XRD), Fourier transform infrared spectroscopy (FT-IR) measurement, scanning electron microscopy/energy dispersive using X-ray analysis (SEM/EDX), and BET/BJH tests were conducted to characterize the crystallinity of the prepared specimens. The XRD analysis was conducted in a 5°–65° 2θ scan range at 0.2°/min scanning rate using PANalytical X’Pert PRO-MPD (DA107 at KBSI Daegu Center). Then, to investigate the quantities of hydroxyapatite and zeolites produced under the different synthesis conditions, the content of the crystalline phases was estimated from XRD diffraction patterns. It should be noted that the reference intensity ratio method, which is suitable method for zeolitic samples synthesized from blast furnace slag, was used to approximate the amount of crystalline phase of the samples [12,26]. SEM images were obtained to investigate the morphology of the crystalline phases in the synthesized specimens. FT-IR measurements (6300FV and IRT5000 device, JASCO, Tokyo, Japan) were conducted in the range of 4000–450 cm^−1^ under a vacuum state using the KBR tablets in the transmission mode. The specific surface areas of the specimens were measured by BET test, and BJH pore volume tests were conducted to investigate the pore size distribution (PSD). EDX analysis was carried out to investigate the elemental composition of the reaction products. Before the EDX analysis, the samples were coated with Pt to enhance the conductivity of the samples, thereby increasing the visibility of the morphologies. It should be noted that the EDX spectra of the samples presented in this study excluded the intensity originated from the presence of Pt for a better comparison with other spectra.

## 3. Results and Discussion

### 3.1. Crystalline Characteristics

**Figure 1:**
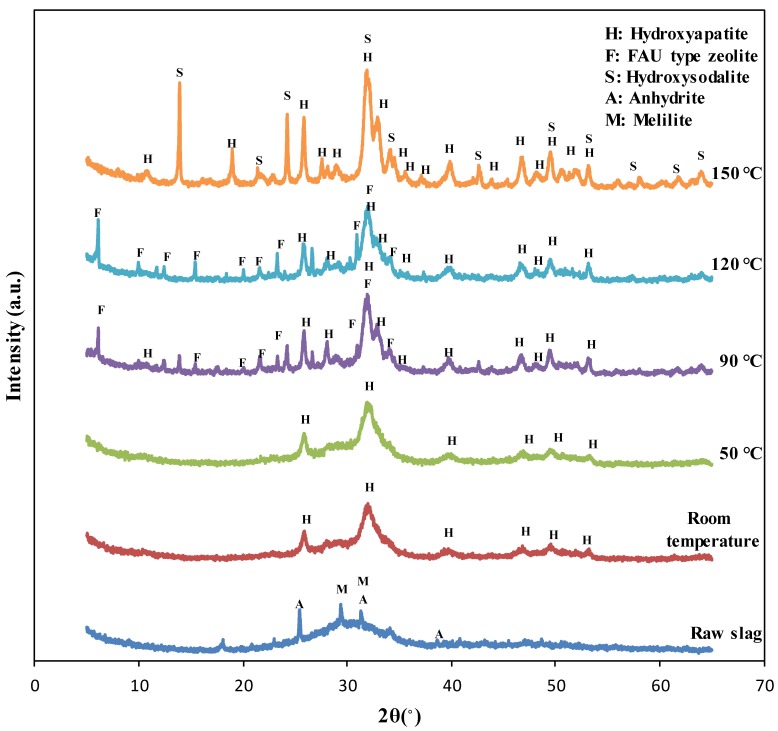
The XRD pattern of raw slag and the specimens synthesized at the different reaction temperature.

The XRD test results of the specimens synthesized at different reaction temperatures are shown in Figure 1. The XRD pattern of the blast furnace slag indicated that the slag included amorphous phases and crystalline peaks of anhydrite (PDF #01-072-0916) and melilite (solid solution of gehlenite, PDF #00-035-0755 and åkermanite, PDF #01-074-0990) [1,12]. The XRD patterns of the specimens synthesized at room temperature and 50 °C showed broader peaks corresponding to hydroxyapatite (PDF #98-003-4457), possibly due to low crystallinity. Alternatively, the peaks of the specimens synthesized at 90 °C, 120 °C, and 150 °C were quite sharp, showing well-developed crystals in these specimens as verified with SEM images. 

The XRD patterns of the specimens synthesized at room temperature and 50 °C showed only hydroxyapatite peaks (PDF #98-003-4457), while those of the specimens synthesized at 90 °C and 120 °C showed faujasite (FAU) type zeolite (PDF #98-008-5622) peaks as well. This indicated that zeolite phases can also be synthesized when the reaction temperature was 90 °C or more. However, the XRD patterns of the specimens synthesized at 150 °C showed the inclusion of hydroxysodalite (PDF #98-016-3788) instead of faujasite because hydroxysodalite can be synthesized at a higher temperature than that to synthesize zeolite [27]. Hydroxysodalite is zeolite-like material, which also possess an affinity to adsorb cations such as heavy metals [12,28].

The crystallinity and quantitative content of the specimens synthesized at different reaction temperatures are listed in Table 2. It was observed that the crystallinity of the specimens fabricated at room temperature and 50°C was similar, meaning that reactivity was the same when the reaction temperature increased to 50°C. The crystallinity of the specimens got doubled when the temperature increased to 90 °C, compared to that of specimens synthesized at lower temperatures. Moreover, the crystallinity of specimens tended to increase with reaction temperature.

Compared to the content in specimens synthesized at reaction temperatures lower than 50 °C, the content of the hydroxyapatite phase increased when the reaction temperature was more than 90 °C. The content of the hydroxyapatite phase did not change significantly at reaction temperatures more than 90°C. Meanwhile, the content of FAU type zeolite or hydroxysodalite increased as the reaction temperature increased. This means that zeolite or zeolite-like phases formed well when the reaction temperature increased beyond 90 °C. On the other hand, the reaction temperature did not significantly influence the quantity of formation of hydroxyapatite, although crystallinity does increase with reaction temperature.

The intensity of the peaks at 603 and 566 cm^−1^, representing PO_4_^3−^ groups in hydroxyapatite, increased with the increase in reaction temperature from room temperature to 90 °C; this peak intensity slightly decreased with further increase in the reaction temperature [1]. This indicates the formation of zeolite crystals in the specimens synthesized above 90 °C. On the other hand, the peak intensities of the SiO_4_^4−^ and PO_4_^3−^ groups decreased with the increase of the reaction temperature to 90 °C. Moreover, the intensities of the CO_3_ peaks (1465–1468 and 1417 cm^−1^) and the dicalcium phosphate dehydrate peak (875 cm^−1^) were highest in the specimen synthesized at room temperature. This seems to be because dicalcium phosphate dehydrate was transformed into hydroxyapatite as the reaction temperature increased. The intensity of two water peaks at 3449–3438 and 1640 cm^−1^ decreased with increasing reaction temperature due to the evaporation of water. It should be noted that the samples were oven dried at the identical temperature used for their synthesis, which resulted in the decrease in the water content in the samples synthesized at high temperatures.

**Figure 2:**
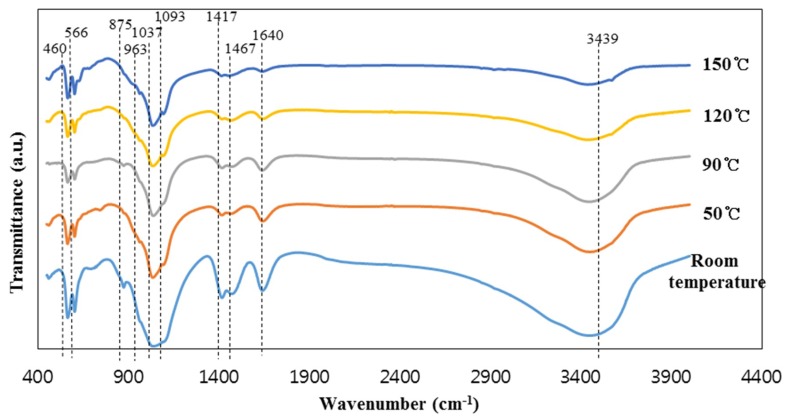
The FT-IR spectra of the materials synthesized at the different reaction temperature.

FT-IR spectra of the specimens synthesized at different reaction temperatures are presented in Figure 2. The spectra of the synthesized specimens showed clear absorption peaks at 1093, 1037, 963, 875, 603, and 566 cm^−1^, which are generally assigned to the vibration of the PO_4_^3−^ groups, although some parts of the PO_4_^3−^ absorption peaks are difficult to discern due to the superposition of the absorption bands with those of the silica group [1,29]. It should be noted that the silica matrix and the hydroxyapatite share many similar vibration modes [1]. This is due to the similarity of the SiO_4_^4−^ and PO_4_^3−^ tetrahedral units [1,29]. The vibration modes are 460 cm^−1^ (Si–O–Si stretch/P–O bending), 963 cm^−1^ (Si–O/P–O symmetric stretch), 1037 cm^−1^ (Si–OH deformation vibration/vibration mode of PO_4_^3−^ group), and 1093 cm^−1^ (Si–O/P–O stretch) [1,29]. However, the four peaks at 1465–1468, 1417, 603, and 565 cm^−1^ are distinctly indicative of hydroxyapatite [1]. The peaks at 1465–1468 and 1417 cm^−1^ are assigned to the carbonate CO_3_^2−^ groups replacing the PO_4_^3−^ in the hydroxyapatite. The peaks of the carbonate groups usually appear in all the hydroxyapatite synthesized in the air [30]. The last two peaks at 603 and 566 cm^−1^ can represent hydroxyapatite formation [1]. The small peak at 875 cm^−1^ is due to HPO_4_^2−^ groups from the reactant, which is dicalcium phosphate dehydrate (CaHPO_4_·2H_2_O), synthesized from blast furnace slag mixed with phosphoric acid without NaOH solution [1,31]. On the other hand, the left two peaks at 3449–3438 and 1640 cm^−1^ represent water [32].

### 3.2. Microstructural Characteristics

BET/BJH test was conducted to investigate the specific surface area and microstructure of the specimens synthesized in this study. The specific surface area and pore characteristics of specimens are presented in Table 3. The test results show that the specific surface area of the specimens synthesized at 50 °C decreased compared to the specimens synthesized at room temperature. However, the specimens synthesized at 90 °C had the largest specific surface area because FAU type zeolite formed, while the specific surface area of the specimens synthesized at 120 °C and 150 °C decreased continuously. This tendency is similar for the cumulative volume of pores from BJH adsorption. The decrease in the specific surface area of the synthesized specimens was possibly attributable to changes in content among hydroxyapatite, FAU type zeolite, and hydroxysodalite (see Table 2). The reason that the specific surface area of the specimens synthesized at 150 °C was lowest seems to be the formation of hydroxysodalite, which has lower specific surface area than that of the FAU type zeolite [12]. The BJH adsorption average pore diameter of the specimens synthesized at 120 °C was the largest and that of the specimens synthesized at 50 °C was the smallest. These results seem to be due to differences in pore distribution of specimens synthesized at different reaction temperatures. Overall the specific surface area decreases as the temperature increases within the temperature range for producing the same crystal phase [33,34]. Moreover, the higher specific surface area can help to improve the adsorption capacity [35,36].

**Figure 3:**
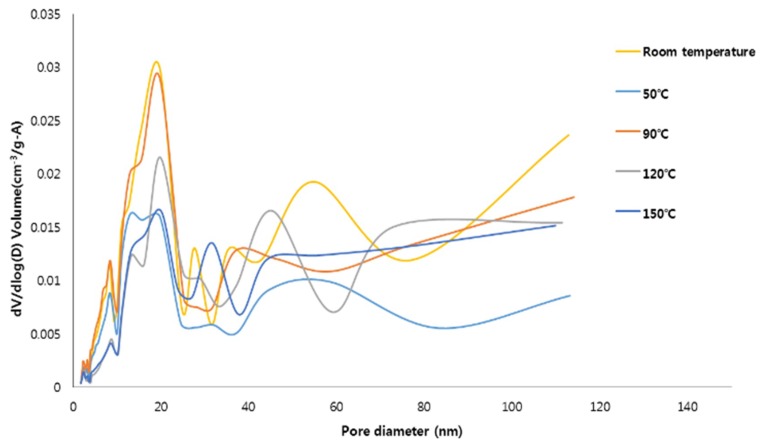
The pore distribution of the materials synthesized at the different reaction temperature.

The pore size distributions (PSD) of the specimens are plotted in Figure 3. For all the specimens, the pores were mostly distributed in the mesoporous range (10 nm and 30 nm). The specimens synthesized at room temperature and 90 °C have a relatively high specific surface area and, compared to the other specimens, were found to contain a large number of pores with sizes between 10 and 30 nm. This pore size range is encouraging because mesoporous materials are advantageous for adsorbing contaminants [12,21,37]. However, it should be noted that the contaminant adsorption capacity of the hydroxyapatite-zeolite could be higher than that of pure hydroxyapatite because zeolite has the higher specific surface area and adsorption capacity than those of hydroxyapatite, implying that zeolite, which can only adsorb cations, and hydroxyapatite can complement each other [1,11].

### 3.3. SEM/EDX Test 

**Figure 4:**
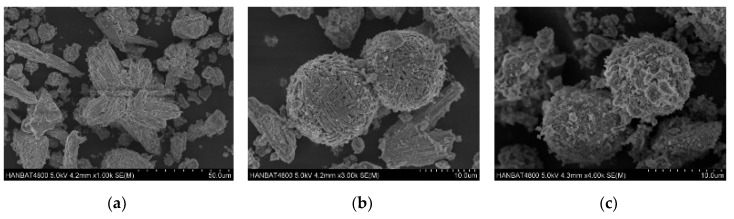
SEM images of (**a**) hydroxyapatite, (**b**) FAU type zeolite, and (**c**) hydroxysodalite.

SEM images of crystals are shown in Figure 4. The hydroxyapatite crystals in the specimens synthesized at 120 °C look like irregular clusters of needles [38] (Figure 4a). The size of the hydroxyapatite crystals was about 300 μm. The crystals with a size of approximately 120 μm and hierarchical pore system are FAU type zeolite, which was observed in the identical specimens [39] (Figure 4b). Hydroxysodalite, which had spherical crystals that aggregate to form agglomerates with a size of approximately 40 μm, was observed in the specimens synthesized at 150 °C, as shown in Figure 4c [28]. 

**Figure 5:**
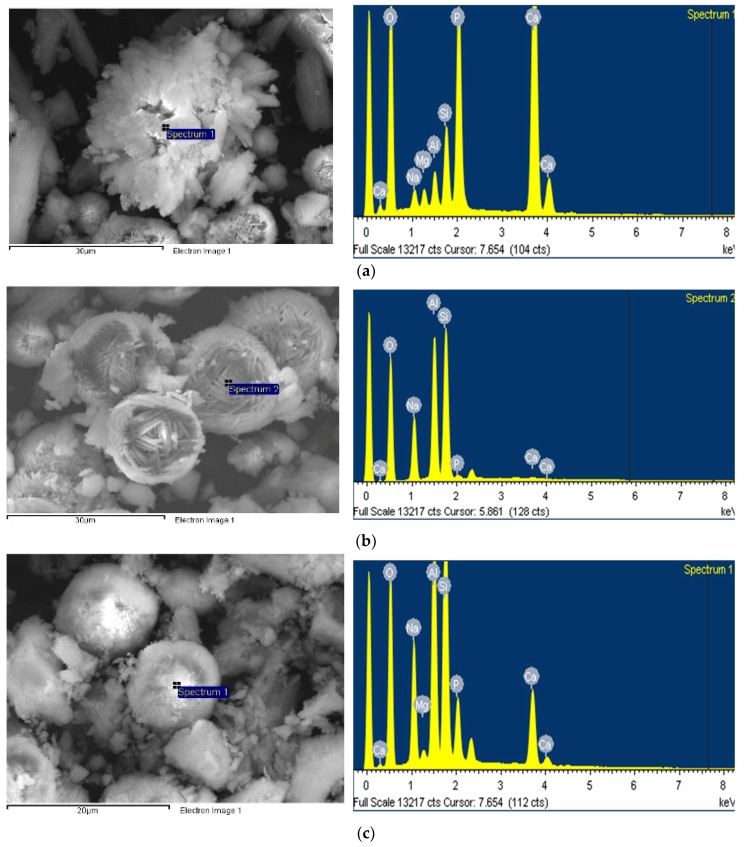
EDX spectra of (**a**) hydroxyapatite, (**b**) FAU type zeolite, and (**c**) hydroxysodalite.

The EDX test results for each crystal are presented in Figure 5. The (Ca + Mg + Na)/P molar ratio of the hydroxyapatite crystals, shown in Figure 5a, corresponding to a stoichiometric composition of 1.68, which means that Ca is partially substituted with Mg and Na components [1]. From an elemental analysis, the FAU type zeolite appears to be a Na-type X-zeolite having a molar ratio of SiO_2_/Al_2_O_3_ = 2.47. The SiO_2_/Al_2_O_3_ ratio of the hydroxyapatite crystals (4.24) was higher than that of the FAU type zeolite crystals. This indicates the Si^4+^ and Al^3+^ ions bound to the hydroxyapatite lattice during hydroxyapatite formation [1].

**Figure 6:**
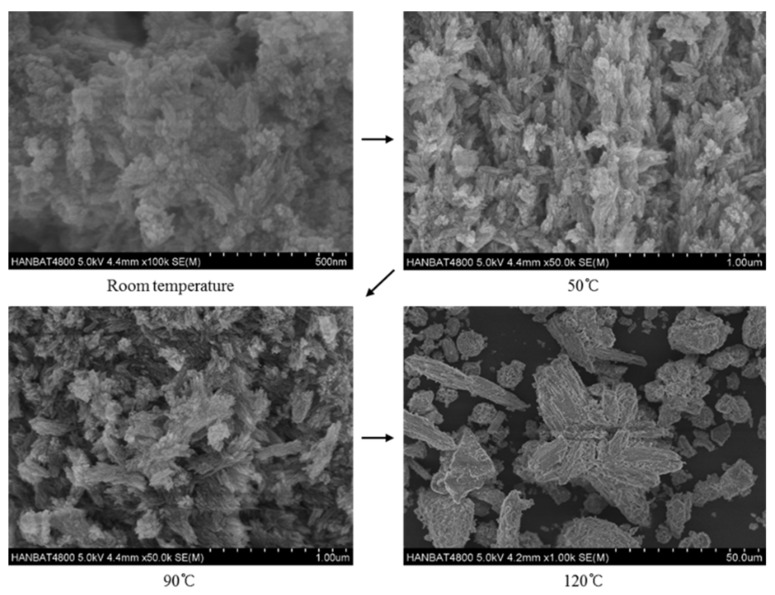
Hydroxyapatite crystalline synthesized at different reaction temperature.

Lastly, the effects of reaction temperature on hydroxyapatite are shown in Figure 6. As the reaction temperature increased, the crystallinity and the size of the hydroxyapatite crystals tended to increase. The hydroxyapatite crystals synthesized at room temperature were about 500 nm in size, and the crystallinity seemed to be low. The hydroxyapatite crystals synthesized at 50 °C showed good growth compared to those synthesized at room temperature; needle-like shapes of specimens can be confirmed in the SEM images [40,41]. In the specimens synthesized at 90 °C, hydroxyapatite crystals had irregular cluster needle shapes, which is similar to the shape of the specimen synthesized at 120 °C, but smaller. The growth of the hydroxyapatite crystal size, as the reaction temperature increase, is consistent with other studies [33,42,43]. This is the reason why the specific surface area decreases as the temperature increases with the same crystal phase (see Table 3) [33,42].

## 4. Concluding Remarks

In this study, the effects of reaction temperature on the synthesis of hydroxyapatite-zeolite from blast furnace slag by the hydrothermal method were investigated. Specimens were synthesized at five different reaction temperatures; followed by characterization through XRD, FT-IR, BET/BJH, and SEM/EDX analyses. The results presented in this study will contribute to broadening the knowledge of physicochemical characteristics of hydroxyapatite-zeolite materials synthesized from blast furnace slag, especially with regard to the reaction temperature. The findings from the present study can be summarized as follows.

1. Up to 50 °C reaction temperature, only hydroxyapatite was synthesized, while FAU type zeolite was formed at 90 °C and 120 °C in addition to hydroxyapatite. With further increase in temperature to 150 °C, hydroxysodalite was synthesized instead of FAU type zeolite along with hydroxyapatite.
2. As the reaction temperature increased, the crystallinity of the specimens tended to increase, and the content of crystalline phases also changed. The content of hydroxyapatite increased with temperature up to 90 °C, while no change was observed with further increment in the temperature.
3. The specimens synthesized at 90 °C had the highest specific surface area of 98.7 m^2^/g, and the specimen synthesized at 150 °C had the lowest specific surface area (51.3) potentially due to the formation of the hydroxysodalite, which has a relatively small specific surface area.
4. The pore size distributions of the specimens prepared at room temperature and 90 °C were largely in the mesoporous range, which is advantageous for adsorbing contaminants.
5. SEM test results showed that hydroxyapatite and FAU type zeolite phases could be clearly observed at the reaction temperature of 120 °C, while hydroxysodalite phase was observed at the reaction temperature of 150 °C instead of FAU type zeolite.

Hence, it can be concluded that 90 °C was the most optimized reaction temperature to synthesize the hydroxyapatite-zeolite using blast furnace slag by the hydrothermal treatment. The results of this study indicate the potential of the synthesized materials to be used as absorbents to remove contaminants [6,7,10,35,38]. However, further study will be needed to investigate the contaminant adsorption capacity of hydroxyapatite-zeolite synthesized by the hydrothermal method.

## 5. Patents

This section is not mandatory, but may be added if there are patents resulting from the work reported in this manuscript.

## Figures and Tables

**Table 1:** Chemical components of blast furnace slag.

Compound	Proportion (wt.%)
Al_2_O_3_	7.50
SiO_2_	18.20
SO_3_	3.10
K_2_O	0.76
CaO	67.60
TiO_2_	0.95
MnO	0.44
Fe_2_O_3_	1.0
NiO	0.04
CuO	0.046
SrO	0.019
ZrO_2_	0.082

**Table 2:** Crystallinity and quantitative content of the materials synthesized at the different reaction temperature.

Reaction Temperature	Amorphous Phase (%)	Crystallinity (%)	Phases	Quantitative Content (%)
Room temperature	64	36	Hydroxyapatite	36
50 °C	66	34	Hydroxyapatite	34
90 °C	37	63	Hydroxyapatite	50
			FAU type zeolite	13
120 °C	43	57	Hydroxyapatite	50
			FAU type zeolite	7
150 °C	30	71	Hydroxyapatite	46
			Hydroxysodalite	25

**Table 3:** BET/BJH results of the materials synthesized at the different reaction temperature.

Reaction Temperature	Room Temperature	50 °C	90 °C	120 °C	150 °C
Specific surface area (m^2^/g)	84.84	69.35	98.74	78.57	51.34
BJH Adsorption cumulative volume of pores (cm^3^/g)	0.28	0.19	0.30	0.19	0.18
BJH Adsorption average pore diameter (nm)	11.30	9.44	9.96	13.37	13.18
